# Version 2.0 of the *Hermes* transposon-based mutagenesis system for prototrophic mutant pool generation in *Candida glabrata*

**DOI:** 10.1128/spectrum.02529-26

**Published:** 2026-09-28

**Authors:** Junhua Wan, Yuru Guo, Yabing Song, Jiaxin Gao

**Affiliations:** 1 State Key Laboratory of Microbial Diversity and Innovative Utilization, Institute of Microbiology, Chinese Academy of Sciences 85387, Beijing, China; CNRS – Inserm – Université Côte d’Azur, Nice, France

**Keywords:** *Hermes *transposon, Prototrophic mutant pool generation, *Candida glabrata*

## Abstract

Although the original *Hermes* transposon mutagenesis system has proven successful for saturated insertional mutagenesis in *Candida glabrata*, its reliance on uracil auxotrophy may limit its applicability under nutrient-restricted host environments. To overcome this limitation, Zaeske et al. (Microbiol Spectr 14:e0084826, 2026, https://doi.org/10.1128/spectrum.00848-26) recently developed a prototrophic alternative that uses cycloheximide (CHX) instead of 5-fluoroorotic acid (5-FOA) to enrich for transposon insertion mutants, generating high-complexity pools. This system provides a valuable resource for investigating the genetic and cellular determinants of colonization and pathogenicity in this important fungal pathogen.

## COMMENTARY

Transposon-based mutagenesis coupled with next-generation sequencing has greatly expanded the functional genomics toolkit for *Candida* species over the past decade. For example, *piggyBac* (*PB*)- and *Ac*/*Ds*-mediated insertional mutagenesis have been successfully applied in a haploid derivative of *Candida albicans*, a leading human fungal pathogen (1–3). More recently, both the *PB* and *Hermes* transposons have been adapted for *C. glabrata*, another important cause of candidiasis (4–8). In parallel, the *PB* transposon has been successfully utilized in the emerging multidrug-resistant human fungal pathogen *Candida auris* (9, 10). These techniques have facilitated the systematic identification of genes and pathways that contribute to clinically relevant traits in *Candida*, including antifungal resistance, stress responses, morphogenesis, and virulence, providing an important foundation for understanding fungal pathogenesis and developing new strategies to combat these infections.

The establishment of *Hermes*-based mutagenesis represented an important technical advance for functional genomics in *C. glabrata*. In an earlier study, Gale et al. adapted the *Hermes* transposon in BG14, a *ura3*Δ derivative of the clinical isolate BG2, and generated highly complex insertion pools containing more than 500,000 unique insertion sites (4). Using this system, they identified hundreds of genes associated with fluconazole susceptibility, including many genes with previously established roles in drug resistance, and performed the first large-scale definition of essential genes in this pathogen. However, the uracil auxotrophy inherent to this system limits its utility in host environments where nutrient availability may differ substantially from that in laboratory media. Indeed, subsequent work demonstrated that the *ura3*Δ mutant exhibits a fitness disadvantage relative to the prototrophic parent strain during gastrointestinal (GI) colonization of mice.

To overcome this potential limitation, Zaeske et al. recently developed an improved *Hermes* system capable of generating insertional mutant pools in prototrophic *C. glabrata* strains by using CHX, rather than 5-FOA, for enrichment (8). Specifically, they introduced a recessive CHX-resistant mutation, *rpl28-Q38E*, into the *C. glabrata* genome and replaced the *URA3* gene on the *Hermes* launchpad plasmid with the dominant, CHX-sensitive *RPL28* gene. Notably, the *rpl28-Q38E* mutation did not affect fitness during colonization of the mouse GI tract, establishing it as a suitable marker for generating mutant pools for genome-scale screening under nutrient-limited host conditions.

Next, Zaeske et al. applied this new system to generate mutant pools in three *C. glabrata* strains from distinct clades (BG2, CBS138, and DPL1021) (8). Analysis of sequencing reads from two BG2r pools revealed that 5-FOA was more efficient than CHX for enrichment, as a small proportion of reads from CHX-enriched pools still mapped to the unexcised launchpad plasmid. More importantly, the comparison of gene representation further revealed the differential abundance of a small number of genes between the CHX- and 5-FOA-enriched pools. Five branched-chain amino acid biosynthesis genes (*ILV1*, *ILV2*, *ILV5*, *ILV3*, *BAT1*) and two functionally redundant protein kinase genes (*PTK1*, *PTK2*) were more abundant in CHX-enriched pools, whereas five adenine biosynthesis genes (*ADE4*, *ADE5*,*7*, *ADE6*, *ADE8*, and *ADE16*) and two negative regulators of RAS-PKA signaling (*IRA1*, *IRA2*) were highly overrepresented in 5-FOA-enriched pools. The biological relevance of this selection bias was supported by functional experiments showing that deletion of either *IRA1* or *IRA2* significantly reduced CHX resistance, as well as by the underrepresentation of known CHX-resistance determinants, including *PDR1*, *CDR1*, and *GAL11-A*, in CHX-enriched pools. This observation highlights an inherent trade-off of CHX-based selection: the selection scheme itself can bias representation of genes involved in CHX resistance. As the underrepresentation of *PDR1* in CHX-enriched pools precluded its assessment in the pooled *in vivo* screen, they directly evaluated the colonization of *pdr1*Δ cells in the mouse GI tract, demonstrating that it promotes *C. glabrata* fitness during gut colonization. Despite this selection bias, CHX-enriched pools retained a complexity comparable to that of 5-FOA-enriched pools, containing more than 587,000 unique insertions and an average insertion density of approximately one insertion every 17 bp after excluding essential and unmappable regions.

Finally, Zaeske et al. compared transposon insertion profiles across chromosomes in the BG2r, DPL1021r, and CBS138r pools, revealing that the CBS138r and DPL1021r pools exhibited markedly higher subtelomeric insertion densities than the BG2r pool, suggesting strain-specific differences in chromatin organization (8). This strain-dependent difference may be associated with a known polymorphism in *SIR3*, which encodes a key regulator of transcriptional silencing. The silencing-promoting *SIR3* variant (N268, I503) is found exclusively in BG2 and closely related clade VII strains, whereas DPL1021 and other clades carry the non-silencing variant (H268, L503).

While the new *Hermes* system offers clear advantages for generating prototrophic mutant pools suitable for host-relevant forward genetic screens, including studies of intestinal colonization, host–pathogen interactions, and the transition from commensalism to invasive infection, its broader utility could be constrained by two technical considerations. First, it requires prior engineering of the strain of interest to introduce the *rpl28-Q38E* mutation before generating a saturation mutant pool. Second, the single-nucleotide A/T footprints left by successive rounds of transposition can result in the accumulation of multiple mutations within a single cell. Consequently, establishing a causal link between a given insertion and the resulting phenotype requires rigorous genetic validation. Nevertheless, these considerations do not diminish the value of the approach; rather, they define important parameters for interpreting genome-scale screens. With appropriate genetic validation, the prototrophic *Hermes* system should substantially expand our ability to dissect *C. glabrata* pathogenesis under host-relevant conditions.
